# Uptake and patterns of PEP use within the context of a dynamic choice HIV prevention model in rural Uganda and Kenya: SEARCH Study

**DOI:** 10.1002/jia2.26450

**Published:** 2025-06-26

**Authors:** James Ayieko, Laura B. Balzer, Colette Aoko, Helen Sunday, Elijah Kakande, Jane Kabami, Catherine Koss, Gabriel Chamie, Moses R. Kamya, Maya L. Petersen, Diane V. Havlir

**Affiliations:** ^1^ Center for Microbiology Research Kenya Medical Research Institute Nairobi Kenya; ^2^ Department of Biostatistics University of California Berkeley California USA; ^3^ Infectious Diseases Research Collaboration Kampala Uganda; ^4^ Department of Medicine University of California San Francisco California USA; ^5^ Department of Medicine Makerere University Kampala Uganda

**Keywords:** biomedical prevention, choice, HIV prevention, post‐exposure prophylaxis, person‐centred, prevention coverage

## Abstract

**Introduction:**

Post‐exposure prophylaxis (PEP) remains underutilized despite being the only prevention option currently available that covers risk after an exposure. We sought to evaluate uptake and patterns of use of PEP among men and women in rural Uganda and Kenya.

**Methods:**

We analysed PEP uptake from three randomized trials enrolling persons aged ≥15 years with HIV risk from antenatal clinics, outpatient departments and community settings from April through August 2021 (NCT04810650). In each trial, participants were randomized to a person‐centred, dynamic choice HIV prevention (DCP) model or standard‐of‐care (SoC) arm. DCP offered choice of biomedical product (oral pre‐exposure prophylaxis [PrEP] or PEP) with an option to switch over time; service location (clinic vs. out‐of‐clinic); testing option (rapid blood‐based test or oral HIV self‐test). The SoC offered HIV prevention services as per in‐country guidelines. In both arms, PEP comprised a 28‐day oral Tenofovir/Lamivudine/Dolutegravir course with HIV testing at start and end of the 28‐day period. We described patterns of and predictors of self‐reported PEP use over the 12 months of follow‐up.

**Results:**

A total of 1232 participants were enrolled, balanced by arm and country. Of the 1147 (93%) who completed at least one survey on self‐reported use of biomedical prevention, the median follow‐up time was 12 months [IQR: 11, 12]. Overall, a total of 104 courses of PEP were dispensed to 59 participants. PEP use was significantly higher among persons enrolled in the DCP arm (relative risk [RR] = 3.30; 95% CI: 1.58−6.91), from Uganda (RR = 3.17; 95% CI: 1.53−6.59), reporting alcohol use (RR = 2.20; 95% CI: 1.30−3.72) and men (RR = 2.08; 95% CI: 1.11−3.91). Of the 59 PEP users, 14 (24%) transitioned to PrEP and 28(47%) used PEP on more than one occasion. Multiple uses of PEP were more common among persons from Uganda versus Kenya (RR = 4.43; 95% CI: 1.10−17.80) and persons enrolled from the community (RR = 4.45; 95% CI: 1.89−10.45) versus clinic. There were no seroconversions reported among PEP users. No serious adverse events were reported.

**Conclusions:**

PEP reaches groups such as men and those who use alcohol who are more likely to benefit from this short‐term prevention modality than PrEP. There is a need to make PEP accessible within a context of person‐centred delivery to optimize its benefits.

## 1 INTRODUCTION

Marked progress has been made in the response against HIV globally [1]. Antiretroviral medications have been a large part of this success both for treatment and prevention [2]. Over the last decade, many countries have rolled out pre‐exposure prophylaxis (PrEP) as part of efforts to reduce incident cases following WHO recommendations [3].

Despite these efforts, 1.3 million (1−1.7m) new HIV acquisitions were reported in 2023 with 630,000 (500,000−820,000) people dying from AIDS‐associated illnesses in the same year [4]. A vast majority of these HIV acquisitions continue to occur in sub‐Saharan Africa with adolescent girls and young women being disproportionately affected (UNAIDS 2024). This gap highlights the need to expand and optimize the use of prevention options to impact HIV incidence.

PrEP access and use is expanding; however, it may not reach or appeal to all persons at risk for acquiring HIV. New injectable long‐acting HIV prevention agents including cabotegravir and lenacapavir provide highly effective, new options for persons at risk for HIV [5, 6, 7]. However, PrEP still requires acknowledgement of HIV risk and willingness to take regimens that start before an exposure. Because these requirements can pose barriers to many individuals, there is a need for additional prevention options [8].

Post‐exposure prophylaxis (PEP) is highly efficacious and recommended in the WHO guidelines [9, 10]; yet, PEP remains underutilized despite being the only prevention option currently available that covers risk after exposure. Past restrictive policies may have limited the use of PEP to those with occupational exposure or sexual assault (including rape) [10] with little use among other high‐risk sexual exposures that would be responsible for a substantial burden of new HIV acquisitions. Our previous work conducted in rural Uganda and Kenya showed that it was feasible to deliver PEP for sexual exposures and highly acceptable in these settings [11]. PEP was well tolerated, and we observed high PEP completion rates with no seroconversion reported among participants in the study.

In this analysis, we move further to evaluate uptake and patterns of PEP use within the context of a dynamic choice HIV prevention (DCP) model as well as the current standard‐of‐care (SoC). Our analysis focuses on PEP use among men and women recruited from diverse settings in rural Uganda and Kenya.

## 2 METHODS

We conducted a secondary analysis of three randomized trials that enrolled men and women aged ≥15 years and reporting HIV risk from antenatal clinic, outpatient department and community settings within the SEARCH‐SAPPHIRE trial in rural Southwestern Uganda and Western Kenya from April to August 2021 (NCT:04810650) [12, 13, 14]. As described elsewhere [12], the DCP arm comprised choice of a biomedical product (oral PrEP or PEP with an option to switch over time), service location (clinic vs. out‐of‐clinic) and testing option (provider‐administered rapid blood‐based test or oral HIV self‐test). Participants randomized to SoC were referred to clinics for HIV prevention services as per in‐country guidelines. PEP regimen comprised oral Tenofovir/Lamivudine/Dolutegravir administered over a 28‐day period in both countries and both study arms. Blood‐based rapid antibody testing was administered prior to PEP start and at the end of the 28‐day period in line with country guidelines.

Using structured surveys administered every 6 months after enrolment, we asked about the use of oral PrEP or PEP. Specifically, for each month in the previous 6 months, we asked the participants if they had swallowed PrEP pills and if they had swallowed PEP pills.

We calculated biomedical HIV prevention coverage, defined as the proportion of follow‐up time covered by a biomedical prevention option. Coverage was calculated among all participants (i.e. irrespective of PEP eligibility), and follow‐up time was censored during months without data on biomedical HIV prevention use. Additionally, among participants with any time covered by a biomedical HIV prevention product, we calculated the proportion attributable to PEP. We emphasize that neither measure captured the proportion of post‐exposure time during which a person eligible for PEP actually took PEP.

We described patterns of PEP use over 12 follow‐up months. To evaluate predictors of PEP use, we used targeted minimum loss‐based estimation (TMLE) [15], a doubly robust approach that generates estimates of risk ratios (RR), instead of odds ratios, for binary outcomes. Among PEP users, we generated an alluvial graph to summarize biomedical prevention use (PEP, PrEP, no product or no data) in 3‐month periods. To understand predictors of multiple uses of PEP, we conducted additional predictor analyses with TMLE. We accounted for clustering by community.

### 2.1 Ethical approval

Ethical approval to conduct the trials was received from the University of California, San Francisco Committee on Human Research, Makerere University School of Medicine Research and Ethics Committee, and the Scientific Ethical Review Unit of the Kenya Medical Research Institute. All participants involved provided written consent to participate in the study.

## 3 RESULTS

The studies enrolled a total of 1232 participants, balanced by country and randomization arm (Table 1). Women comprised nearly three‐quarters of all participants, who were recruited from antenatal clinics (32%), the outpatient department (33%) and the community (35%). The median age of participants was 26 [IQR: 21, 35] years and 41% of all participants were aged 15−24 years. A total of 281 (23%) reported alcohol use.

**Table 1:** Characteristics of participants at enrolment, among those who used PEP and among those who used PEP multiple times

	Overall	Used PEP	Used PEP multiple times
	*N* = 1232	*N* = 59	*N* = 28
**Arm**			
Dynamic choice HIV prevention	612 (50%)	45 (76%)	24 (86%)
Standard‐of‐care	620 (50%)	14 (24%)	4 (14%)
**Country**			
Kenyan	612 (50%)	15 (25%)	2 (7%)
Ugandan	620 (50%)	44 (75%)	26 (93%)
**Sex**			
Women	888 (72%)	33 (56%)	14 (50%)
Men	344 (28%)	26 (44%)	14 (50%)
**Recruitment setting**			
Antenatal clinic	400 (32%)	6 (10%)	0 (0%)
Outpatient department	403 (33%)	23 (39%)	5 (18%)
Community	429 (35%)	30 (51%)	23 (82%)
**Age**, median [Q1, Q3]	26 [21, 35]	27 [22, 38]	31 [22, 41]
Age 15–24 years	506 (41%)	24 (41%)	8 (29%)
Age 25 years+	726 (59%)	35 (59%)	20 (71%)
**Marital status**			
Single (never married)	310 (25%)	18 (31%)	9 (32%)
Married/cohabitating	872 (71%)	39 (66%)	19 (68%)
Divorced/separated/widowed	48 (4%)	2 (3%)	0 (0%)
**Occupation**			
Farmer	448 (36%)	27 (47%)	15 (56%)
Student	169 (14%)	8 (14%)	3 (11%)
Shopkeeper/market vendor	113 (9%)	6 (10%)	1 (4%)
Manual labour/construction	59 (5%)	2 (3%)	2 (7%)
Transportation	29 (2%)	4 (7%)	0 (0%)
Bar/hotel/restaurant	35 (3%)	0 (0%)	0 (0%)
Fishing/fishmonger	16 (1%)	0 (0%)	0 (0%)
**Alcohol use**	281 (23%)	22 (37%)	11 (39%)
**Pregnant** (women only)	178 (20%)	5 (16%)	0 (0%)
**Circumcised** (men only)	202 (59%)	14 (56%)	7 (50%)

*Note*: Unless noted, metrics are in *N* (column %).

Abbreviations: PEP, post‐exposure prophylaxis; PrEP, pre‐exposure prophylaxis.

Of the 1232 participants, 1147 (93%) completed at least one survey on self‐reported use of biomedical prevention. Among those surveyed, 990 (86%) completed both surveys; the median follow‐up time was 12 months [IQR: 11, 12], and biomedical HIV prevention coverage was 32.3% on average (median = 9.3%).

Overall, a total of 104 courses of PEP were dispensed, accounting for 7.2% of covered time. These courses were dispensed to 59 participants (Table 1). Of the 59 courses of PEP dispensed, 45 (76%) were in the DCP arm and the remaining 14 (24%) in the SoC arm. DCP participants were significantly more likely to use PEP (RR: 3.30 [95% CI: 1.58−6.91]; *p*<0.01). Ugandan participants had higher uptake of PEP than Kenyan participants (RR: 3.17 [1.53−6.59]; *p*<0.01). Persons reporting any alcohol used were significantly more likely to use PEP (RR: 2.20 [1.30−3.72]; *p*<0.01), but there were no differences between younger (15−24 years) and older (25+ years) persons (RR: 0.98 [0.61−1.57]; *p* = 0.92). Men were more than twice as likely to use PEP as compared to women (RR: 2.08 [1.11−3.91]; *p* = 0.02). As compared to recruitment from clinical sites, participants recruited from the community tended to be more likely to use PEP (RR: 1.85 [0.77−4.43]; *p* = 0.17).

**Figure 1:**
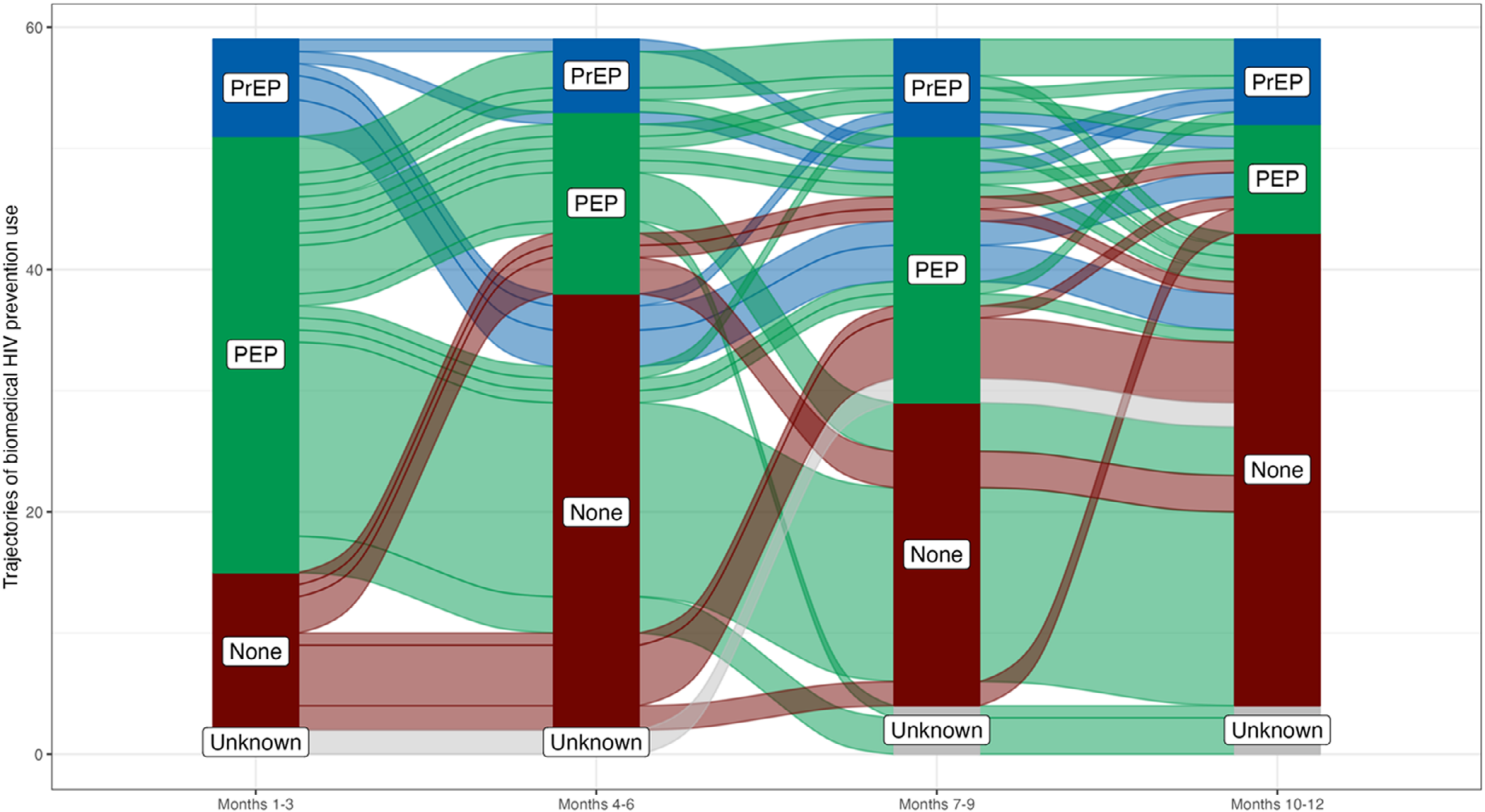
Alluvial plot of the trajectories of biomedical HIV prevention use among participants who used PEP. Abbreviations: PEP, post‐exposure prophylaxis; PrEP, pre‐exposure prophylaxis.

Among PEP users, Figure 1 provides a visual summary of the trajectories of biomedical prevention use in 3‐month windows over follow‐up. This alluvial plot demonstrates that choice and use of biomedical prevention are highly dynamic. Over the 12 months of follow‐up, 10 PEP users transitioned to oral PrEP immediately after finishing a PEP course, while a total of 14 participants transitioned to PrEP sometime after finishing a PEP course. Furthermore, 12 PEP users were previously on PrEP.

Of the 59 PEP users, 28 participants used PEP on more than one occasion (Table 1). One was more likely to use PEP on more than one occasion if they were from Uganda versus Kenya (RR: 4.43 [1.10−17.80]; *p* = 0.04) or recruited from the community versus clinical sites (RR: 4.45 [1.89−10.45]; *p*<0.01). Use of multiple PEP courses were not significantly different by trial arm (RR: 1.87 [0.76−4.59]; *p* = 0.17), alcohol use (RR: 1.09 [0.66−1.81]; *p* = 0.74), age (RR: 0.58 [0.26−1.29]; *p* = 0.18) or sex (RR: 1.27 [0.71−2.27]; *p* = 0.41).

There were no seroconversions reported among PEP users. No serious adverse events were reported.

## 4 DISCUSSION

PEP remains a crucial HIV prevention option that merits increasing attention to realize its potential to reduce HIV incidence and maintain clients in prevention programmes. A portion of our study population only chose and used PEP over the study period despite the availability of other options. These are individuals who would likely have lacked biomedical prevention had PEP not been available, and instead received prevention for high‐risk periods. This finding confirms patterns seen in other studies [16] and further underscores the importance of provision of particular client‐preferred options regardless of the volume of uptake if the effectiveness of HIV prevention options is to be optimized.

Beyond this benefit, it is important to consider that PEP is a unique avenue for the expansion of HIV prevention coverage time when persons are at risk of HIV acquisition. Globally, PEP use has been low, this has largely been driven by low levels of awareness of clients and providers, inaccessibility and stigma [17, 18]. Sensitization, provider training and increased access of PEP, therefore, become crucial elements in expanding PEP use. Other studies have shown low use of this effective option among groups at elevated risk such as men‐who‐have‐sex‐with‐men due to other barriers such as the 28‐day oral pill burden [19]; long‐acting PrEP agents could overcome this limitation.

PEP remains an important entry point to other HIV prevention options. We observed transitions from PEP to other options based on risk assessment and client preference over time. PEP should, therefore, not be viewed as stand‐alone but as part of a comprehensive HIV prevention package. The WHO HIV prevention guidelines envision these transitions for individuals selecting options over time [10]. However, periods of transition present the risk of disengagement. We posit that person‐centred provider–guided delivery of PEP among other HIV prevention options, would keep persons engaged in the care system as shown by the higher uptake and engagement in our DCP arms compared to the SOC. Our person‐centred provider‐guided DCP model was designed to ensure client–provider engagement even in periods when clients felt that they were not at risk or preferred not to select an option [12–14]. This approach facilitated a safe start and stoppage of prevention options over time while maintaining open communication to allow for future selection of other options. HIV prevention models should, therefore, be deliberate in optimizing engagement through approaches that are person‐centred.

Certain groups in our studies were more likely to utilize PEP. Men were more likely to use PEP than women. It is possible that this is due to women having higher risk aversion than men in social risk‐taking [20, 21], or that men are more hesitant to commit to a daily PrEP pill, or that men engaged in unplanned sex more than women. We further noted that those who took alcohol were more likely to use PEP consistent with prior reports of high‐risk behaviour during alcohol use [22, 23]. This finding delineating certain groups as being at elevated‐risk helps to highlight areas that can be intervened upon. Innovative programmes have been successfully designed to modify behaviour, influence choices and improve the uptake of health initiatives [24, 25, 26]. Behavioural initiatives have also been used to reduce alcohol use and impact health outcomes [27]. All these approaches should be integrated in designing and delivering effective HIV prevention approaches. Settings of delivery also determine uptake as shown by higher uptake in the community supporting the WHO recommendation on the expansion of delivery of PEP to community settings [10].

**Figure 2:**
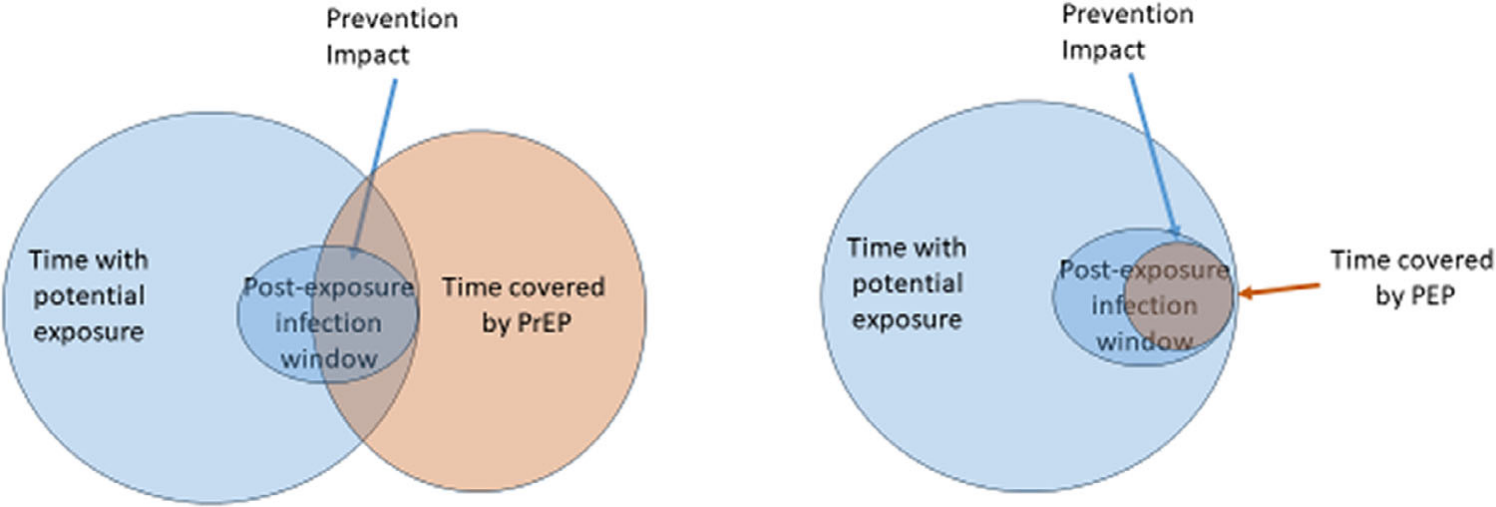
Venn diagrams on post‐exposure prophylaxis (PEP) and pre‐exposure prophylaxis (PrEP) covered time with potential exposure versus potential prevention impact.

We observed a number of PEP repeat users in our study. Within the context of choice and patient‐centred provider‐guidance, repeated use of PEP is not a “failure.” Previous studies among groups at elevated risk have shown repeated use as well as intention for repeat use [18, 28] similar to what was observed in our study. Indeed, repeat use could be seen as an extremely effective approach for aligning coverage with true risk. Conceptually, coverage of “true” risk and potential impact of PEP is markedly different from coverage with pre‐exposure options. PEP covered time is highly focused on post‐exposure window when an HIV acquisition can occur. The Venn diagrams included here (Figure 2) help conceptualize this idea. If time with potential exposure is the circle in blue, it is preferred that prevention options overlay this area to the greatest extent possible to have the desired effect. Whereas for PrEP one would take medication continuously for prevention, the medication would cover even periods without risk. PEP, on the other hand, follows a high‐risk exposure and for this brief period of time is likely to have high prevention coverage and impact for the known risk. This would further suggest that when comparing with pre‐exposure options, the magnitude of the time covered may not be as important as the events targeted by PEP. Therefore, the magnitude of coverage time is not a measure of the impact or success of PEP use. As metrics of measuring HIV prevention continue to be refined, PEP should be understood as distinct and unique from other prevention options used prior to risk exposure.

The study had limitations. First, our assessment of PEP use relied on self‐report, which may be subject to recall bias. However, our structured surveys have been previously validated using objective biomarkers in the same study settings [13, 14]. Second, our survey evaluated any PEP use in each month over a 6‐month period, limiting the granularity of the data. Additionally, we did not collect data on PEP completion nor did we report exposure or reason for PEP choice. However, these data limitations should not induce bias in our analyses to assess predictors of PEP use or patterns over time. Qualitative research would help explore mechanisms of choice of PEP over PrEP given risk exposure. Finally, our trials did not compare PEP use in the context of on‐demand PrEP for men, dapivirine ring for women or injectable PrEP which are options that may appeal to some individuals.

## 5 CONCLUSIONS

PEP reaches groups who are more likely to benefit from this short‐term prevention modality than PrEP, such as men and those who use alcohol, and contributes to averting new HIV acquisitions. PEP remains an important prevention option to be assessed even as PrEP options continue to expand. There is an urgent need to train providers on choice models and make PEP accessible within a context of person‐centred delivery to optimize its benefits.

## COMPETING INTERESTS

The authors have no conflicts of interest to disclose.

## AUTHORS’ CONTRIBUTIONS

All authors participated in the conduct of the study. JA, LBB, CA, HS, EK, JK, GC, MRK and DVH designed and participated in the implementation of the study. JA, CK, DVH, MLP and LBB evaluated data integrity. JA and DVH drafted the first version of the manuscript which was reviewed and approved by all authors.

## FUNDING

Research reported in this manuscript was supported by the National Heart, Lung, and Blood Institute (NHLBI), the National Institute of Allergy and Infectious Diseases (NIAID), and the National Institute of Mental Health (NIMH) and co‐funded under award number U01AI150510.

## DISCLAIMER

The content is solely the responsibility of the authors and does not necessarily represent the official views of the NIH.

## Data Availability

The data that support the findings of this study are available on request from the corresponding author. The data are not publicly available due to privacy or ethical restrictions.
